# Genetic Correlates as a Predictor of Bariatric Surgery Outcomes after 1 Year

**DOI:** 10.3390/biomedicines11102644

**Published:** 2023-09-27

**Authors:** Panayotis K. Thanos, Colin Hanna, Abrianna Mihalkovic, Aaron Hoffman, Alan Posner, John Butsch, Kenneth Blum, Lesley Georger, Lucy D. Mastrandrea, Teresa Quattrin

**Affiliations:** 1 Behavioral Neuropharmacology and Neuroimaging Laboratory on Addictions, Department of Pharmacology and Toxicology, Clinical Research Institute on Addictions, Jacobs School of Medicine and Biosciences, University at Buffalo, Buffalo, NY 14203, USA; cshanna2@buffalo.edu (C.H.); amihalko@buffalo.edu (A.M.); 2 Department of Psychology, University at Buffalo, Buffalo, NY 14203, USA; 3 Department of Surgery, Methodist Hospital Medical Center, Dallas, TX 75208, USA; aaronhoffman@mhd.com; 4 Department of Surgery, Jacobs School of Medicine and Biomedical Sciences, University at Buffalo, Buffalo, NY 14203, USA; arposner@buffalo.edu (A.P.); jbutsch@kaleidahealth.org (J.B.); 5 Division of Nutrigenomics, SpliceGen, Therapeutics, Inc., Austin, TX 78701, USA; drd2gene@buffalo.edu; 6 Department of Psychiatry, Wright State University Boonshoft School of Medicine and Dayton VA Medical Center, Dayton, OH 45435, USA; 7 Division of Addiction Research & Education, Center for Exercise Sports & Global Mental Health, Western University Health Sciences, Pomona, CA 91766, USA; 8 The Kenneth Blum Behavioral & Neurogenetic Institute, LLC., Austin, TX 78701, USA; 9 Institute of Psychology, ELTE Eötvös Loránd University, 1075 Budapest, Hungary; 10 Centre for Genomics and Applied Gene Technology, Institute of Integrative Omics and Applied Biotechnology (IIOAB), Nonakuri, Purba Medinipur 721172, West Bengal, India; 11 Department of Molecular Biology, Adelson School of Medicine, Ariel University, Ariel 40700, Israel; 12 Department of Natural Sciences and Mathematics, D’Youville University, Buffalo, NY 14201, USA; georgerl@dyc.edu; 13 UBMD Pediatrics, JR Oishei Children’s Hospital, University at Buffalo, Buffalo, NY 14203, USA; ldm@buffalo.edu (L.D.M.); quattrin@buffalo.edu (T.Q.)

**Keywords:** bariatric surgery, obesity, addiction, genetics, psychosocial, *DRD2* gene, GARS, Reward-Deficiency Syndrome, dopamine homeostasis, behavioral addiction

## Abstract

This study analyzed genetic risk assessments in patients undergoing bariatric surgery to serve as a predictive factor for weight loss parameters 1 year after the operation. Thirty (30) patients were assessed for Genetic Addiction Risk Severity (GARS), which analyzes neurogenetic polymorphisms involved in addiction and reward deficiency. Genetic and psychosocial data collected before the operation were correlated with weight loss data, including changes in weight, body mass index (BMI), and percent of expected weight loss (%EWL). Results examined correlations between individual gene risk alleles, 1-year body weight data, and psychosocial trait scores. Spearman’s correlations revealed that the *OPRM1* (rs1799971) gene polymorphism had significant negative correlation with 1-year weight (r_s_ = −0.4477, *p* < 0.01) and BMI (r_s_ = −0.4477, *p* < 0.05). In addition, the *DRD2* risk allele (rs1800497) was correlated negatively with BMI at 1 year (r_s_ = −0.4927, *p* < 0.05), indicating that one risk allele copy was associated with lower BMI. However, this allele was positively correlated with both ∆Weight (r_s_ = 0.4077, *p* < 0.05) and %EWL (r_s_ = 0.5521, *p* < 0.05) at 1 year post-surgery. Moreover, the overall GARS score was correlated with %EWL (r_s_ = 0.4236, *p* < 0.05), ∆Weight (r_s_ = 0.3971, *p* < 0.05) and ∆BMI (r_s_ = 0.3778, *p* < 0.05). Lastly, Food Cravings Questionnaire (FCQ) scores were negatively correlated with %EWL (r_s_ = −0.4320, *p* < 0.05) and ∆Weight at 1 year post-surgery (r_s_ = −0.4294, *p* < 0.05). This suggests that individuals with a higher genetic addiction risk are more responsive to weight loss treatment, especially in the case of the *DRD2* polymorphism. These results should translate clinically to improve positivity and attitude related to weight management by those individuals born with the risk alleles (rs1800497; rs1799971).

## 1. Introduction

Among adults worldwide, obesity is a steadily growing problem [1,2,3,4,5,6,7,8,9,10,11]. In 2008, this global health issue impacted approximately 1.5 billion adults [12]. By 2016, this number climbed to 1.9 billion adults worldwide [13]. By the year 2030, 1.35 billion individuals are projected to be overweight, and obese adult numbers are projected to reach 573 million individuals [14]. If this issue remains neglected, these numbers are projected to reach 2.16 billion overweight individuals and 1.12 billion obese individuals by 2030 [14].

There is evidence to support that obesity and eating disorders are related to psychiatric comorbidities [15,16,17,18,19,20,21,22,23,24]. Among Brazilian obese patients, binge eating disorders were found to correlate with depression and suicidal thoughts [25]. Additionally, patients seeking bariatric weight loss surgery often suffer from various affective and psychological disorders including anxiety, depression, and body image dissatisfaction [26].

One challenge in weight management science is that most treatments for obesity are considered unsustainable over time [27,28]. Bariatric surgery is considered an optimal weight loss method for individuals unable to achieve efficient results from typical, non-surgical weight loss interventions [29]. The two common types of bariatric surgeries include gastric sleeve and bypass surgery (or laparoscopic sleeve gastrectomy and Roux-en-Y gastric bypass, respectively). One clinical study found that after 7 years, gastric sleeve surgery resulted in a 47% weight loss, gastric bypass surgery resulted in a 55% weight loss, and both surgeries resulted in an improved quality of life [30].

However, this procedure can pose post-operative behavioral risks such as increased rates of alcohol abuse [31,32,33]. In fact, many substance and non-substance behavioral addictions (such as gambling disorders) tend to increase after obesity operations [34]. Interestingly, common genetic liability to alcohol consumption problems (ACP) and suicide attempts (SA) were significantly correlated with all impulsive personality traits (r_s_=  0.2–0.53, *p*  <  0.002), and the largest correlation was with lack of premeditation, though supplementary analyses suggested that these findings were potentially more influenced by ACP than SA [35,36]. It is noteworthy that in a genome-wide association study among veterans with a history of attempted suicide, a strong pan-ancestry signal at the dopamine receptor D2 locus (*p* = 1.77 × 10^−7^) was identified and subsequently replicated in a large, independent international civilian cohort (*p* = 7.97 × 10^−4^)^7^ [37].

Identifying individuals who may be at risk for behavioral addictions can influence and personalize post-surgical intervention methods for those with obesity. This can potentially maximize benefits and likelihood of surgical success. Assessments for at-risk patients can occur in a couple of different fashions. First, psychological assessments can be utilized to discern which patients might be struggling with body image issues and affective disorders, thus influencing the course of pre-surgical preparations and post-operative behavioral follow-ups [26,29,38,39].

In addition to psychological screenings, an individual’s genetic makeup can be observed [40,41,42,43] to highlight a propensity towards behavioral addictions [44], giving clinicians further opportunities to tailor interventions and maximize the likelihood of the operation’s success. Genetic addiction risk has been previously described to identify genetic polymorphisms (alleles) known to play a role in addiction, compulsive behaviors (such as overeating) [45,46], vulnerability to pain [47], and behavioral/conduct disorders [48]. A partial summary of these genes and their polymorphisms, locations, and risk alleles are shown in Table 1. Briefly, these genes are known to play a role in mesolimbic neurotransmission: modulating neurotransmitter systems such as GABA receptors, serotonin transporters, mu-opioid receptors, multiple neurotransmitter enzymes, and, most importantly, receptors and transporters in dopaminergic neurotransmission [49]. Together, alterations in their neurogenetic markers establish a framework for epigenetic behavioral expressions known as Reward Deficiency Syndrome (RDS) [50]. The candidate genes relating to RDS have been thoroughly investigated in hundreds of studies. A meta-analysis of 74,566 case-controlled subjects showed a significant risk of alcohol-use disorder in the presence of *DRD2*, *DRD3*, *DRD4*, *DAT1*, *COMT*, *OPRM1*, and *5HTT* polymorphisms [51].

We presently examined the role of specific psychosocial and genetic factors and their association with weight data outcomes in patients undergoing bariatric surgery. The objective of the present study was to examine this pre-operative data and identify its predictive ability in the trajectory of post-operative outcomes. Genetic and psychosocial data were correlated with post-operative body weight data 1 year after surgery.

## 2. Materials and Methods

### 2.1. Subjects

Initially, 70 bariatric surgery candidates were consulted at Kaleida Health Bariatric Center in Buffalo, NY. Of these, 34 subjects provided initial informed consent.

Among these participants, the mean age was 47 (SD = 12.33). A total of 10.3% of these participants were males and 89.7% were females. The mean height of these participants was 165 cm (SD = 7.38) and the mean pre-operative bodyweight was 118 kg (SD = 20.76). The mean BMI was 43 (SD = 6.02). Of the individuals that reported race (*n* = 27), 85.19% were white, 11.11% were black or African, and 3.7% were Hispanic. Pre-operative bloodwork of these participants included the following measures: glucose mean 102.62 mg/dL, SD = 31.28. Triglyceride mean = 144.04 mg/dL SD = 82.45 and cholesterol mean = 193.2 mg/dL, SD = 38.33.

Exclusion criteria included pregnant women, prisoners, and those with significant cognitive or neurological impairments. Data were collected on medical history, comorbidities and other conditions treated, and weight history. More than half of the sample reported a childhood history of obesity. A total of 48% of subjects reported alcohol use (M ≤ 1 drinks per week). Cigarette use was reported in 1 patient. A total of 42% of patients reported orthopedic pain. A total of 39% of patients had depression. A total of 81% of patients experienced sleep apnea. Data were collected at 1-year post-surgery follow-up visits for 30 subjects. Lack of follow ups due to the COVID-19 pandemic resulted in a smaller than anticipated sample size.

### 2.2. Surgery

All patients received either laparoscopic sleeve gastrectomy or Roux-en-Y gastric bypass surgery. A total of 23 individuals received laparoscopic sleeve gastrectomy, and 7 individuals received Roux-en-Y gastric bypass.

### 2.3. Data Collection

Parameters relating to health pre- and post-surgery (1 year) were collected from electronic health records (2021–2022). Change in weight and BMI from 1 year after surgery were calculated.

### 2.4. Psychosocial Questionnaires

Patients were given surveys in both paper in digital formats. The surveys can be seen in Table 2. These validated scales were used to evaluate psychosocial data related to obesity and eating habits. These reports included: nutrition (Eating Attitudes Test-26 (EAT-26) [53]; Food Cravings Questionnaire—Trait Reduced (FCQ-TR) [54]; Eating Expectancies Inventory (EEI) [55]; food addiction (modified Yale Food Addiction Scale 2.0 (mYFAS 2.0) [56]; binge-eating disorder symptoms (Weight-Influenced Self-Esteem Questionnaire (WISE-Q) [57]; depression and anxiety (Difficulties in Emotion Regulation Scale) (DERS) [58]; Center for Epidemiologic Studies Depression Scale (CESDS) [59], and chronic stress and life quality (Chronic Stress Index (CSI) [60]; sleep (Pittsburgh Sleep Quality Index (PSQI) [61]. This methodology was utilized as previously described [62].

### 2.5. Genetic Addiction Risk Severity (GARS)

The GARS assay (Geneus Health, San Antonio, TX, USA) is a genetic test used to evaluate eleven gene polymorphisms known to be involved in motivation and reward. This test is commonly used to predict RDS, a propensity for addictive behaviors (such as eating disorders), and a tendency towards substance abuse. Prior to surgery, cheek swab samples were collected from subjects and processed according to previously published protocol [63]. PCR amplification was used to isolate DNA, which was then analyzed for polymorphisms in genes: *DRD1*, *OPRM1*, *DRD2*, *DRD3*, *DRD4*, *COMT*, *DAT1*, *DRD4-R*, *GABRB3*, *HTTLPR*, and *MAOA* [45,49]. Geneus Health in San Antonio, Texas provided analysis and results. Individual risk scores were calculated as previously described [47,49,62,64].

### 2.6. Statistical Analysis

Data were assessed and visualized using GraphPad Prism software 8.1.2 (Dotmatics, San Diego, CA, USA). Spearman’s rank correlations were analyzed for ∆BMI and ∆Weight 1 year after surgery date. GARS risk alleles were correlated with ∆BMI, ∆Weight, and psychosocial scores. Tukey’s HSD test, Sidak’s test was performed post hoc (when applicable) for significant ANOVA outcomes.

### 2.7. Ethics

This study was approved by and complied with the Institutional Review Board of the University at Buffalo (#IRB00003126). All subjects were fully informed about the nature of the study, and all provided informed consent.

## 3. Results

### 3.1. Baseline Demographic Characteristics

Participants (*n* = 30) were recruited from the Bariatric Program at Kaleida Health, which is designated as a Comprehensive Center under the Metabolic and Bariatric Surgery Accreditation and Quality Improvement Program. This study was approved by the IRB at the University at Buffalo. Participants were predominantly female and Caucasian, with >50% reporting a childhood history of overweight/obesity. Of these subjects, 74% underwent vertical sleeve gastrectomy. The COVID-19 epidemic prevented us from obtaining psychosocial questionnaires and follow-up data in 10 participants.

### 3.2. Psychosocial and GARS Data

A majority of subjects disclosed symptoms of depression, issues in sleep quality, and food addiction and cravings. These reports are in agreement with previous psychosocial studies on obesity [56,59,61,65]. The Yale Food Addiction Scale (mYFAS) results were lower than anticipated [66,67]. The summarized psychosocial scores (previously reported) [62] can be seen in Table 3.

GARS results were categorized as homozygote (two copies of the risk allele), heterozygote (one copy of the risk allele), or low risk (no copies of the risk allele). Homozygote alleles were most present in the *MAO* and *DRD1* genes. No subjects were homozygous for risk alleles in genes *OPRMI*, *DRD4* (rs761010487), and *DAT1f*. A GARS score above or equal to 7 indicates a high risk for addiction and RDS. In total, 76% of subjects were categorized as high-risk. Previous studies have shown that a high GARS score is correlated with an increased risk for alcohol abuse [45,51,68,69,70].

### 3.3. Risk Allele Correlates

**Figure 1:**
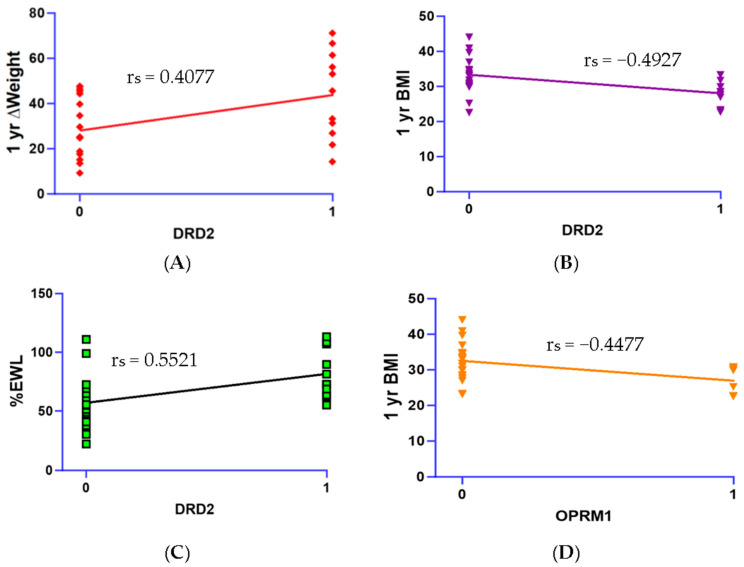
Scatterplots visualizing correlations between SNPs and body weight data. (**A**) Positive correlations between single *DRD2* SNPs and 1 yr ∆Weight (r_s_ = 0.4077, *p* < 0.05). (**B**) Negative correlations between single *DRD2* SNPs and 1 yr BMI (r_s_ = −0.4927, *p* < 0.05). (**C**) Positive correlation between *DRD2* SNPs and %EWL (r_s_ = 0.5521, *p* < 0.05). (**D**) Negative correlation between *OPRM1* SNP and 1 yr BMI (r_s_ = −0.4477, *p* < 0.05).

**Figure 2:**
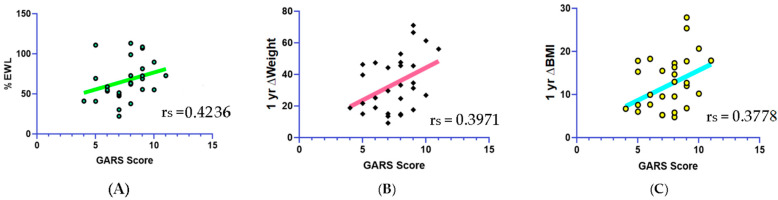
Correlations between overall GARS score. (**A**) %EWL (r_s_ = 0.4236, *p* < 0.05). (**B**) 1 yr ∆Weight (r_s_ = 0.3971, *p* < 0.05). (**C**) 1 yr ∆BMI (r_s_ = 0.3778, *p* < 0.05).

**Figure 3:**
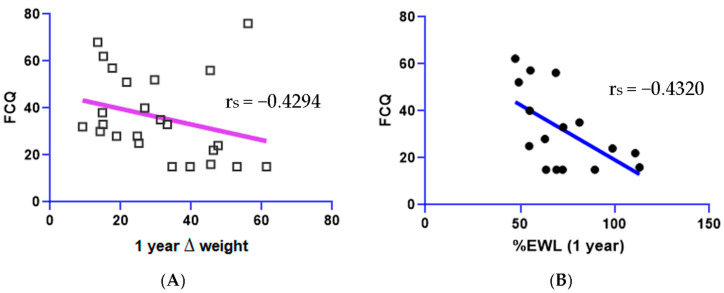
Negative correlations between (**A**) FCQ and (**B**) 1 yr ∆Weight (r_s_ = −0.4294, *p* < 0.05) and %EWL (r_s_ = 0.4320, *p* < 0.05).

Spearman’s correlations revealed that the *OPRM1* showed a significant negative correlation with 1-year weight (r_s_ = −0.4477, *p* < 0.01, 95% CI: −0.7052, −0.08583) and BMI (r_s_ = −0.4477, *p* < 0.05, 95% CI: −0.7052, −0.08590). *DRD2* was negatively correlated with BMI at 1 year (r_s_ = −0.4927, *p* < 0.05, 95% CI: −0.7331, −0.01429), positively correlated with ∆Weight (r_s_ = 0.4077, *p* < 0.05., 95% CI: 0.03711, 0.6797), and positively correlated with %EWL (r_s_ = 0.5521, *p* < 0.05, 95% CI: 0.2219, 0.7687) at 1 year post-surgery. The results of these correlations with SNPs are shown in Figure 1. The overall GARS score was correlated with %EWL (r_s_ = 0.4236, *p* < 0.05, 95% CI: 0.05629, 0.6899), ∆Weight (r_s_ = 0.3971, *p* < 0.05, 95% CI: 0.02445, 0.6729), and ∆BMI (r_s_ = 0.3778, *p* < 0.05, 95% CI: 0.001782, 0.6603) (Figure 2). Lastly, FCQ scores were negatively correlated with %EWL (r_s_ = −0.4320, *p* < 0.05, 95% CI: −0.7176, −0.022) and ∆Weight at 1-year post surgery (r_s_ = −0.4294, *p* < 0.05, 95% CI: −0.7160, −0.01879) (Figure 3).

**Figure 4:**
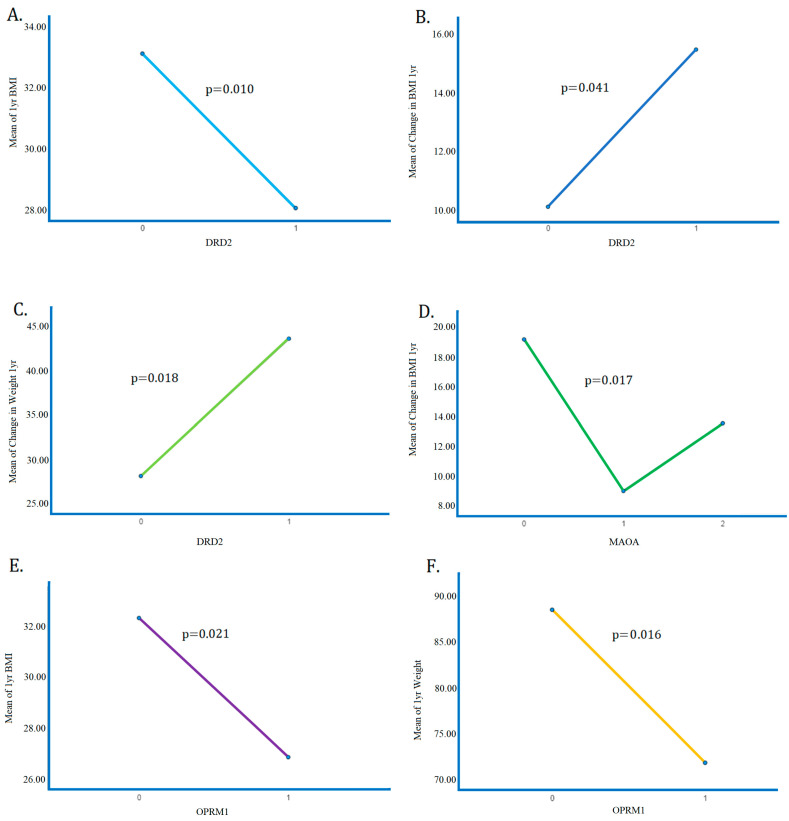
ANOVA results detailing the significant differences in means of (**A**) 1-year BMI between subjects with 0 and 1 copy of the *DRD2* gene (*p* = 0.010); (**B**) ∆BMI between subjects with 0 and 1 copy of the *DRD2* gene (*p* = 0.041); (**C**) ∆Weight between subjects with 0 and 1 copy of the *DRD2* gene (*p* = 0.018); (**D**) ∆BMI between subjects with 0 and 1 copies of the *MAOA* gene (*p* = 0.017). Note: difference in mean insignificant between 0–2, 1–2 copies of the *MAOA* gene. (**E**) 1-year BMI between subjects with 0 and 1 copy of the *OPRM1* gene (*p* = 0.021). (**F**) 1-year weight between subjects with 0 and 1 copy of the *OPRM1* gene (*p* = 0.016).

A one-way ANOVA was performed to compare means of weight, BMI, ∆Weight, and ∆BMI between the different SNP expression values (0, 1, or 2). The Tukey HSD post hoc test was performed where relevant. There is a significant difference in 1 year BMI (*p* = 0.010), ∆BMI (*p* = 0.041), and ∆Weight (*p* = 0.018) between 0 and 1 *DRD2* risk allele copy. There is a significant difference in 1-year BMI (*p* = 0.021) and 1-year weight (*p* = 0.016) between 0 and 1 copy of the *OPRM1* risk allele. (Subjects with two copies of the *DRD2* risk allele and the *OPRM1* risk allele were not represented in the sample.) There is also a significant difference in ∆BMI (*p* = 0.017) among the different SNP expression values of the *MAOA* risk allele. Tukey HSD post hoc tests indicate that there is a significant difference (*p* = 0.017) in ∆BMI between 0 and 1 copy of the *MAOA* risk allele, but not between 0 and 2 copies or 1 and 2 copies. These results are visualized in Figure 4.

A post hoc power analysis was conducted using G*Power 3.1 [71] to test the correlation using a two-tailed test, an alpha of 0.05, a moderate effect size (r = 0.40), and a sample size of *n* = 29. Results showed that the achieved power was 0.59.

## 4. Discussion

These results reflect a beneficial response to weight loss surgery in individuals with indicators of high genetic addiction risk. Those with higher GARS scores show greater changes in weight, %EWL, and change in BMI 1 year after bariatric surgery. Our ANOVA results indicated a significant difference in mean weight change between individuals with 0 and 1 copy of the *MAOA* gene, with 1 copy resulting in lower average weight change. The ANOVA and Spearman’s correlations revealed a significant improvement in weight parameters in patients with 1 copy of the *OPRM1* and the *DRD2* gene.

The *DRD2* gene, located on chromosome 11q23, is the most widely studied gene in neuropsychiatry [72,73,74,75,76,77,78,79,80,81,82,83,84,85,86,87,88,89,90,91,92]. The A1 risk allele is associated with various substance and non-substance addictions [69,93,94]. Carriers of this risk allele show a decreased availability of dopamine D2 receptors [95,96], which can result in D2 receptor super-sensitivity [97], increasing severity of alcoholism [98,99], obesity [28], and addiction relapse [97].

A1 allelic presence is related to many facets of obesity [7,43,51,55,56]. *DRD2* variants were associated with BMI in individuals seeking weight loss treatment [73]. Parental obesity, postpubescent onset, and a preference for carbohydrates have all been linked to the A1 obese phenotype [98]. The A1 allelic presence was found in 45.2% of 73 nonalcohol- and nondrug-abusing obese subjects. This presence was observed in 51.5 subjects with a history of parental obesity. Carbohydrate preferers displayed 64.3% of this allelic presence. Even fat distribution was found to have a hereditary component [100,101].

We believe that the results of this clinical study are likely the result of D2 modulation. At first glance, it may seem contradictory that individuals with a genetic susceptibility to addictive eating and obesity would have such a positive response to bariatric surgery. However, compliance to addiction treatment has been observed in alcoholics with the A1 polymorphism [102]. Bromocriptine treatment (dopamine agonist therapy) proved to produce the most significant attenuations in craving and anxiety amongst A1 carrier alcoholics [102]. This genotype was associated with reductions in body weight, fat mass, and BMI after among subjects who underwent resistance training and calorie restriction for weight loss. In addition, *DRD2* polymorphisms are correlates of longitudinal obesity mitigation in Chinese children and adolescents [103]. Moreover, carriers of the *DRD2* A1 allele with diminished D2 receptor availability show a positive association between caudate response and change in weight [94].

We speculate that the surgical intervention directly modulated the dopaminergic reward system. It is known that D2 availability can decrease with overstimulation from overeating [104,105,106] and D2 striatal receptor availability is significantly decreased in cases of severe obesity [28]. These results suggest that surgery bypassed D2 super sensitivity and decreased the wanting mechanism in these obese patients.

There is in fact some evidence pointing to an upregulation/normalization of D2 receptors after bariatric surgery [28,107,108,109,110]. In a preclinical autoradiography study, rats on a chronic high-fat diet became obese and showed decreased D1 and D2 receptors in the nucleus accumbens and striatum. Rats who were given a high-fat diet and Roux-en-Y gastric bypass surgery showed no difference in DA receptor levels when compared to restricted diet rats, suggesting that striatal and nucleus accumbens dopamine systems can be normalized after bariatric surgery [109].

This phenomenon is observable in clinical studies as well. Striatal D2 and D3 availability was assessed in morbidly obese women after Roux-en-Y gastric bypass surgery [108]. At first, striatal availability of these receptors decreased at baseline and remained after 6 weeks. After 2 years, however, the availabilities of these receptors increased and improved body weight data were observed [108]. Additionally, among five female subjects undergoing this same bariatric procedure, significant weight loss was observed and D2 receptor availabilities increased in the anterior and posterior putamen and caudate nucleus, and in the ventral striatum [107].

The Mu-Opioid Receptor is known to modulate reward processing, motivation, and hedonic behaviors [111]. This gene is commonly assessed to help determine genetic addiction risk. However, its role in eating disorders and obesity has only been slightly investigated. One study assessing *ORM1* polymorphism, rs2281617 (different from presently observed polymorphism) linked genetic data with feeding behavior, adiposity, and amygdala volume in 598 adolescents [112]. BOLD fMRI results showed that this polymorphism was associated with higher amygdala volume, which correlated negatively with fat intake. It is believed that the *OPRM1* gene and variations of amygdalar volumes modulate dietary intake of fat [112].

Though there are fewer studies relating the *OPRM1* gene to obesity, Positron Emission Tomography (PET) studies using the receptor agonist radiotracer 11C-carfentanil have specified the role of this receptor in obesity and eating behaviors. Multiple studies have found that OPRM1 availability is negatively related to obesity and food addiction [113,114,115,116]. First, there is evidence to suggest that familial obesity is related to decreased availability of the OPRM1 [114]. OPRM1 availability has also been associated with eating habits as indicated by the Dutch Eating Behavior Questionnaire [114]. This study revealed decreased OPRM1 availability correlated with an increase in external eating. Subjects with decreased receptor availability showed an increased likelihood of responding to palatable food cues by eating [114].

Karlsson et al. observed the dynamics of obesity and the *OPRM1* gene. In this study, 13 morbidly obese women underwent [(11)C]carfentanil PET scans. When compared to controls, decreased availability of OPRM1 was observed in the ventral striatum, insula, and thalamus. BMI was associated negatively with OPRM1 availability [116]. Brain responses to palatable foods occur in non-obese individuals as well. A BOLD fMRI study detected activation in the amygdala, ventral striatum, and hypothalamus after subjects were shown palatable food cues. OPRM1 availability was negatively associated with this fMRI reward response [111].

The *MAOA* gene encodes for enzymes responsible for breaking down monoamine neurotransmitters, including serotonin and dopamine [117,118]. Variations of this gene play a role in psychiatric disorders including substance use disorders and conduct/antisocial personality disorders [117,119]. Variations in this gene are associated with disease comorbidities because of the enzyme’s direct actions on dopamine levels [120].

The evidence linking this gene prompts further investigation. One study investigating *MAOA* and *COMT* genotypes in obese subjects compared to controls found no significant relation between the *MAOA* genotype and obesity [121]. Another study assessing the same gene and similar repeat sequences of interest to our own (3.5R, 4R). The results of this study reflected a strong significance of the *MAOA* genotype on body weight and BMI [118]. In a group of young Portuguese adults, body fat and the *MAOA* 3R genotype were correlated in men only [122]. The significant difference in mean change of BMI after 1 year of bariatric surgery was only observed between individuals having 0 or 1 copy of the risk alleles, with 1 copy having the less favorable outcome lower average changes in BMI. Mean differences between 0 and 2 or 1 and 2 copies were found to be insignificant. This may be related to subtle changes in DA levels among this genotype.

## 5. Limitations

A small sample size due to lack of follow ups during COVID-19 pandemic can be considered a limitation of this study. Genetic and psychosocial data are cofactors of post-surgical results, while epigenetics and other variables were not the focus of this study.

## 6. Conclusions

This novel comparison between genetic and psychosocial factors predicted outcomes following bariatric surgery. These results suggest that individuals with specific genetic alleles and psychosocial scores are significantly correlated with weight loss and outcomes following bariatric surgery. Specifically, patients carrying the *DRD2* A1 allele (rs1800497) and the mu-opioid allele (1799971) significantly correlated with greater weight loss following bariatric surgery. Understanding these results should clinically translate to the patient providing additional positivity and as such augmented attitude based on genetic and psychosocial information. This report is the second part of a longitudinal study observing the genetic and psychosocial effects on bariatric surgery outcomes [62]. A summary of the present findings along with previous data can be seen in Table 2. Future studies will track these same data at longer time intervals after bariatric surgery. Notes of recidivism, including for substance and non-substance addictive behaviors, will be closely monitored as well. These subjects will continue to be monitored for long-term outcomes beyond the present study.

## Figures and Tables

**Table 1:** GARS panel. Table adopted from Blum et al., 2020 [52].

Gene	Polymorphism	Location	Risk Allele(s)
Dopamine D1 Receptor DRD1	rs4532 SNP	Chr5	A
Dopamine D2 Receptor DRD2	rs1800497 SNP	Chr11	A
Dopamine D3 Receptor DRD3	rs6280 SNP	Chr3	C
Dopamine D4 Receptor DRD4	rs1800955 SNP	Chr11	C
	48 bases Repeat VNTR	Chr11, Exon 3	7R, 8R, 9R, 10R, 11R
Catechol-O-Methyltransferase COMT	rs4680 SNP	Chr22	G
Mu-Opioid Receptor OPRM1	rs1799971 SNP	Chr6	G
Dopamine Active Transporter DAT 1	40 bases Repeat VNTR	Chr5, Exon 15	3R, 4R, 5R, 6R, 7R, 8R
Monoamine Oxidase A MAOA	30 bases Repeat VNTR	Chr X, Promoter	3.5R, 4R
Serotonin Transporter SLC6A4 (5HTTLPR)	43 bases Repeat INDEL/VNTR plus rs25531 SNP	Chr 17	LG, S
GABA(A) Receptor, Alpha-3 GABRB3	CA-Repeat DNR	Chr 15 (downstream)	181

**Table 2:** Summary of previous and present findings: Genetic and psychosocial correlates of bodyweight data after Bariatric Surgery at 6 months and 1 year post-operation. Data from 6 months post-operation were previously reported [62].

	6 Months	12 Months
∆BMI and a mean % excess weight loss	(56 ± 13.8%)	% EWL (*p* < 0.05), ∆Weight (*p* < 0.05), and ∆BMI (*p* < 0.05).
GARS scores above 7	76% of subjects GARS significantly correlated (increases) with ∆ weight and ∆ BMI	76% of subjects correlated with ∆ weight and ∆ BMI.
GARS scores	significantly correlated (increases) with ∆ weight and ∆ BMI	
The *DRD2* risk allele		Positively correlated (increases) with ∆Weight (*p* < 0.05), and positively correlated (increases) with % Expected Weight Loss (EWL) (*p* < 0.05)-negatively correlated (decreases) with BMI at 1 year (*p* < 0.05). -one copy of the risk allele was associated with lower BMI.
The *COMT* risk allele	negative correlation (decreases) with EEI scores *p* < 0.05) and PSQI scores (*p* < 0.05)	
*GABRB3* risk allele	correlated positively (increases) with EEI (*p* < 0.01) and FCQ scores *p* < 0.01)	
*OPRM1* risk allele	positive correlation (increases) with the DERS score (*p* < 0.05)	Spearman’s correlations showed a significant negative correlation (decreases) with 1-year weight (*p* < 0.01) and BMI (*p* < 0.05)
The *DRD2* risk allele		-negatively correlated (decreases) with BMI at 1 year (*p* < 0.05). -one copy of the risk allele was associated with lower BMI. -positively correlated (increases) with ∆Weight (*p* < 0.05), and positively correlated (increases) with % EWL (*p* < 0.05)
Food Cravings Questionnaire (FCQ) scores		negatively correlated (decreases) with %EWL (*p* < 0.05) and ∆Weight (*p* < 0.05).
	CONCLUSIONS These data support the potential benefit of a personalized medicine approach, including genetic testing and psychosocial trait questionnaires when counseling patients with obesity considering bariatric surgery.	CONCLUSIONS Based on previous work, carriers of the *DRD2* risk allele (rs1800497) are significantly more compliant with pharmacological treatment, and spearmen correlations had the highest compliance to behavioral therapeutics, thus lower BMI compared to non-carriers.

**Table 3:** Psychosocial Questionnaire Results as previously reported by Thanos et al., 2023 [62].

Eating Attitudes Test-26	Total: 14.9 (8.1)
Food Cravings Questionnaire—Trait Reduced (FCQ-T)	- Domain Control: 2.3 (1.17) - Thoughts: 2.1 (1.23) - Plans: 2.5 (1.57) - Emotions: 2.4 (1.33) - Cues: 2.7 (1.54)
Eating Expectancies Inventory	- Manage Negative Affect: 2.91 (2.02) - Pleasurable and Useful as a Reward: 3.62 (2.23) - Feeling Out of Control: 3.12 (2.11) - Enhances Cognitive Competence: 2.69 (1.82) - Alleviates Boredom: 3.35 (2.23)
Modified Yale Food Addiction Scale 2.0	Mean Symptom Count (SD): 1.32 (1.23) No Food Addiction (%): 61 Mild (%): 31 Moderate (%): 4 Severe (%): 4
Weight-Influenced Self Esteem Questionnaire	M (SD): 1.6 (1.3)
Difficulties in Emotion Regulation Scale—Short Form	Total Mean (SD): 33.81 (10.96) - Total w/o Awareness: 27.5 (10.52) - Awareness: 6.35 (2.46) - Clarity: 4.61 (1.80) - Goals: 7.58 (3.88) - Impulse: 4.23 (2.3) - Non-acceptance: 5.65 (2.67) - Strategies: 5.38 (2.89)
Center for Epidemiological Studies Depression Scale	Total Score (Mean, range): 12.7, 0–35 No Depression (%): 69 Mild Depression (%): 8 Probable Depression (%): 23
Chronic Stress Index	Perceived Everyday Unfair Treatment (Mean Score): 1.8 Major Negative Life Events in Past Year: 1.13
Quality of Life Enjoyment and Satisfaction Questionnaire	M (SD): 3.24 (0.89)
Pittsburgh Sleep Quality Index	M (SD): 8.0 (3.74)

Summary of scored outcomes from self-report psychosocial questionnaires completed by patients prior to surgery (*n* = 26). Mean score totals and subscale scores for each inventory.

## Data Availability

Data are available from the corresponding author upon reasonable request.
